# Announcement

**Published:** 2015-03-06

## Ground Water Awareness Week — March 8–14, 2015

CDC is collaborating with the National Ground Water Association to highlight National Ground Water Awareness Week, March 8–14, 2015. Water is essential for life. However, many persons are not aware that much of the water they use flows from below ground to the surface to public water systems and private wells. The National Ground Water Association uses this week to stress ground water’s importance to the health and well-being of humans and the environment (1).

The majority of public water systems in the United States use ground water as their primary source, providing drinking water to almost 90 million persons in nearly 34 million households (2,3). An additional 34 million persons in approximately 13 million households use private wells (3,4).

Ground water in the United States generally is considered safe to use. However, ground water is susceptible to naturally occurring or man-made contamination. Contamination can be from arsenic; pesticides; industrial, agricultural, and resource extraction wastes; and municipal sewage as a result of failures in treatment or improper disposal into the environment. The exposure to contaminants at harmful levels can lead to acute and chronic illness (5,6).

The U.S. Environmental Protection Agency has implemented new regulations to provide increased protection against microbial pathogens in public water systems that use ground water sources (7). Private ground water wells (serving fewer than 25 persons or having less than 15 connections) might not be regulated but nonetheless must be properly maintained by well owners to ensure that the water remains free from harmful chemicals and pathogens.* Resources are available from state and local health departments and nonprofit organizations to help homeowners protect their ground water.†

## Footnotes

* Additional information available at http://www.cdc.gov/healthywater/drinking/private/wells/index.html.

† Additional information available at http://www.cdc.gov/healthywater/drinking/training-education.html#webtraining.
